# Identifying potential (re)hemorrhage among sporadic cerebral cavernous malformations using machine learning

**DOI:** 10.1038/s41598-024-61851-4

**Published:** 2024-05-14

**Authors:** Xiaopeng Li, Peng Jones, Mei Zhao

**Affiliations:** 1 Department of Neurology, The First Affiliated Hospital of Henan University, Kaifeng, China; 2 Independent Researcher, Xinyang, Henan China; 3 Department of Neurology, The First Affiliated Hospital of Nanchang University, No. 17 Yongwai Street, Nanchang, 330006 Jiangxi China

**Keywords:** Cerebral cavernous, Malformations, Intracerebral hemorrhage, Machine learning, 4-Elements model, Outcome prediction, Diseases, Neurology

## Abstract

The (re)hemorrhage in patients with sporadic cerebral cavernous malformations (CCM) was the primary aim for CCM management. However, accurately identifying the potential (re)hemorrhage among sporadic CCM patients in advance remains a challenge. This study aims to develop machine learning models to detect potential (re)hemorrhage in sporadic CCM patients. This study was based on a dataset of 731 sporadic CCM patients in open data platform Dryad. Sporadic CCM patients were followed up 5 years from January 2003 to December 2018. Support vector machine (SVM), stacked generalization, and extreme gradient boosting (XGBoost) were used to construct models. The performance of models was evaluated by area under receiver operating characteristic curves (AUROC), area under the precision-recall curve (PR-AUC) and other metrics. A total of 517 patients with sporadic CCM were included (330 female [63.8%], mean [SD] age at diagnosis, 42.1 [15.5] years). 76 (re)hemorrhage (14.7%) occurred during follow-up. Among 3 machine learning models, XGBoost model yielded the highest mean (SD) AUROC (0.87 [0.06]) in cross-validation. The top 4 features of XGBoost model were ranked with SHAP (SHapley Additive exPlanations). All-Elements XGBoost model achieved an AUROCs of 0.84 and PR-AUC of 0.49 in testing set, with a sensitivity of 0.86 and a specificity of 0.76. Importantly, 4-Elements XGBoost model developed using top 4 features got a AUROCs of 0.83 and PR-AUC of 0.40, a sensitivity of 0.79, and a specificity of 0.72 in testing set. Two machine learning-based models achieved accurate performance in identifying potential (re)hemorrhages within 5 years in sporadic CCM patients. These models may provide insights for clinical decision-making.

## Introduction

Cerebral cavernous malformations (CCM), mostly caused by loss-of-function of mutations genes^1^, are vascular lesions of the brain with a risk of causing intracerebral hemorrhage (ICH)^1–3^. CCM show a familial or sporadic form^4^, and also could be detected after radiation therapy^5^, almost 20% of CCM found with multiple locations^6,7^. These CCM-related ICH mainly caused headaches, seizures, impaired consciousness, and focal neurological deficits^8^. A meta-analysis with 7 patient cohorts demonstrated that a 5-year ICH risk for CCM was 15.8% using reported standards^9^. As the most feared complication, symptomatic (re)hemorrhage is the primary aim for CCM management^4^, especially repetitive hemorrhage leading to being disabled and fatal^10,11^. Most previous studies focused on identifying risk factors of ICH among CCM patients^7,12–14^. A report with a dataset containing 731 CCM patients followed up from 2003 to 2018 based on Cox proportional hazards model showed that prior ICH and brainstem localization were associated with a higher risk of (re)-hemorrhage^15^. Using this large and invaluable dataset, machine learning models also could be constructed to detect potential (re)-hemorrhage with several clinical records of CCM patients. Identifying the potential (re)bleeding in advance among CCM patients and initiating prompt treatment, such as surgical resection, conservative treatment^1,4^ or long-term antithrombotic therapy use^16–19^, is essential for CCM management.

However, the established machine learning model for detecting potential (re)hemorrhage among CCM patients is still lacking. The prediction models based on machine learning algorithms showed robust performance in various areas including medical events^20–23^. Thus, we suppose that machine learning algorithms might make it possible to yield accurate prediction models, even providing limited medical information about CCM patients.

The present study aimed to develop and validate prediction models that could distinguish sporadic CCM patients of potential (re)hemorrhage from those without risk of (re)hemorrhage within 5 years. Here, we report machine learning models with comparatively high predictability for identifying potential (re)hemorrhage within 5 years, which may provide insights for clinical decision-making for the treatment of sporadic CCM patients.

## Methods

### Participants

This study included a dataset of 731 sporadic CCM patients in the data platform Dryad, the collection of which was approved by university institutional review and written consent was acquired from all patients^15,24^. These consecutively admitted patients were prospectively followed up 5 years from January 1, 2003, to December 31, 2018^15^. Hemorrhage during registration and occurrence of (re)hemorrhage in follow-up were evaluated by reported standards, and 64% completeness of follow-up with a high censoring rate was due to surgical treatment^15^.

### Study design and feature selection

We selected 517 sporadic CCM patients and 12 features in this dataset, which include: age at diagnosis, sex, supratentorial CCM, CCM at brain stem, CCM at infratentorial nonbrain stem, CCM volume, developmental venous anomaly (DVA), hypercholesterolemia, hypertension, diabetes, prior ICH, (re)hemorrhage during follow-up within 5 years. For patients with prior ICH, CCM volume was measured via the sum of CCM lesion and hemorrhage lesion^15^. Patients during follow-up without (re)hemorrhage receiving surgical treatment and those with missing information about surgery information in follow-up were excluded. Missing values in features of hypercholesterolemia, diabetes, and hypertension were imputed using multiple imputation by chained equations (MICE) with the aid of python module miceforest^25^.

### Machine learning algorithm and dealing with imbalanced data

Support vector machine (SVM), one robust supervised machine learning method, is used for analyzing datasets for classification and regression^26,27^. Extreme gradient boosting (XGBoost) is an ensemble learning algorithm based on decision trees^28^, showing accurate performance in the medical field^29,30^. Stacked generalization, often termed as stacking, super learning, or stacked regression^31,32^, combines multiple base classifiers with a final classifier aiming at reducing biases. Stacking is a common method to ensemble various algorithms into a powerful learner^31^. For our stacking model, we implemented decision trees^33^, random forests, gradient boosted decision trees (GBDT)^34^, SVM, multi-layer perceptron^35^, and k nearest neighbors^36^ as the base estimators, and logistic regression as the final estimator.

Among all included 517 sporadic CCM patients, 76 patients occurred (re)hemorrhage during follow-up, yielding imbalance. Dealing with imbalanced data for machine learning algorithms is challenging in academia and industry^37^. Random under-sampling has been adopted to reduce the majority class^20,21^, to aid the algorithm in identifying the minority class. In the training and validation cohort, we applied random under-sampling to reduce the size of sporadic CCM patients without (re)hemorrhage.

### Model development and feature importance

The dataset was randomly split into the training and validation cohort (80%) and the testing cohort (20%). The prediction models were built with the aid of the efficient tool scikit-learn (version 1.0.2) and other modules (pandas, numpy, matplotlib). The hyperparameters were tuned to maximize the area under the receiver operating characteristic curves (AUROC) with the aid of GridSearchCV in the training and validation cohort. We trained three models using three repeats of five-fold stratified cross-validation. Models performance, including precision, recall, and F-score, was also calculated in the process of cross-validation. The search space for hyperparameters and the chosen values for all models are shown in Supplementary Table S1. Other parameters were set as default values.

We explored the importance ranking of features based on the XGBoost model interpreted by SHAP (SHapley Additive exPlanations). The 4-Elements model was built using the top 4 features (CCM volume, prior ICH, CCM at brain stem, age at diagnosis).

### Model performance in testing cohort

To validate the performance of the XGBoost models, we calculated the AUROC and the area under the precision recall curve (PR-AUC). The evaluation metrics, including sensitivity, specificity, positive predictive value (PPV), negative predictive value (NPV), positive likelihood ratio, and negative likelihood ratio, were also computed.

### Statistical analysis

The normally distributed continuous variable was analyzed via a two-sided t-test, whereas the Mann–Whitney test was conducted for nonnormally distributed continuous variables. Categorical data were performed via χ^2^ test including continuity correction in case of low frequencies. Statistical significance was set at p < 0.05 (two-sided). Statistical analyses were conducted with the use of SAS software, version 9.4 (SAS Institute Inc).

### Ethical considerations

The dataset of sporadic CCM patients is sourced from the open data platform Dryad. Standard protocol and registrations of patients were approved by the institutional review board of Duisburg-Essen University (review board identification 14-5751-BO and 19-8662-BO)^15^. The written consent was also acquired from all patients^15^. All procedures of this study involving human participants were in accordance with the ethical guidelines of the declaration of Helsinki.

## Results

### Baseline characteristics

**Figure 1:**
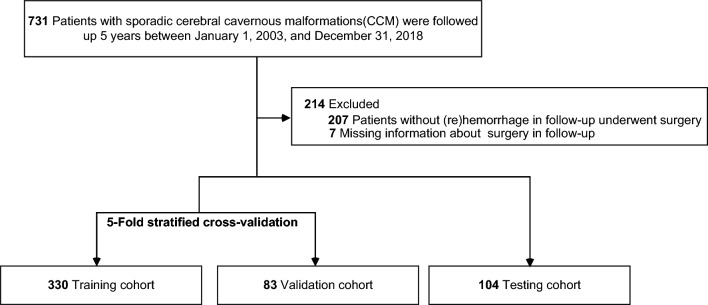
Flow diagram of study.

A total of 517 sporadic CCM patients were included in this study cohort (330 female patients [63.8%], mean [SD] age at diagnosis, 42.1 [15.5] years), among whom 76 patients (14.7%) experienced (re)hemorrhage during 5-year follow-up. The dataset was randomly assigned to the training set and the testing set. The baseline features of the two groups are shown in Table 1. The flow diagram of the modeling has been illustrated in Fig. 1.

**Table 1:** Baseline characteristics of the patients cohort.

	Training and validation cohort (n = 413)	Testing cohort (n = 104)	P value
Age at diagnosis, years; mean (SD)	41.5 (15.8)	44.6 (14.2)	0.065
Female (%)	263 (63.7)	67 (64.4)	0.888
Supratentorial CCM (%)	257 (62.2)	64 (61.5)	0.897
CCM at Infratentorial nonbrain stem (%)	40 (9.7)	9 (8.7)	0.748
CCM at brain stem (%)	126 (30.5)	32 (30.8)	0.959
CCM volume, cm^3^; median (IQR)	0.54 (0.18–2.00)	0.72 (0.17–1.90)	0.918
Associated DVA (%)	168 (40.7)	39 (37.5)	0.554
Prior ICH (%)	175 (42.4)	45 (43.3)	0.869
Hypertension (%)	89 (21.5)	30 (28.8)	0.114
Hypercholesterolemia (%)	27 (6.5)	13 (12.5)	0.042^a^
Diabetes (%)	15 (3.6)	4 (3.8)	1.000
Outcome: (re)hemorrhage (%)			
Yes	62 (15)	14 (13.5)	0.690
No	351 (85)	90 (86.5)	

*IQR* interquartile range, *CCM* cerebral cavernous malformations, *DVA* developmental venous anomaly, *ICH* intracerebral hemorrhage.

^a^P < 0.05.

### Comparison of model's performance in cross-validation

**Figure 2:**
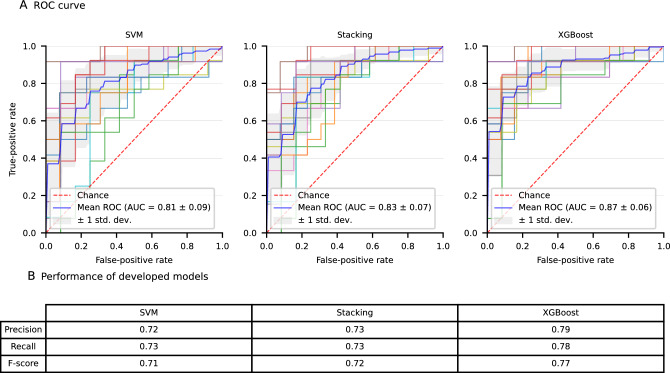
The Performance of Models in Identifying the Potential (Re)hemorrhage in Cross-Validation. (**A**) The receiver operating characteristic (ROC) curves of machine-learning based models using 3 repeats of fivefold stratified cross-validation. (**B**) Representative performance of 3 machine learning models. *AUC* area under the curves, *CCM* cerebral cavernous malformations, *SVM* support vector machine, *Stacking* stacked generalization, *XGBoost* extreme gradient boosting.

3 prediction models were developed using 11 features of sporadic CCM patients. For the evaluation of metrics of models, sporadic CCM patients who occurred (re)hemorrhage during follow-up were treated as true positives whilst those without risk of bleeding were considered to be true negatives. ROC curves and the performance of three prediction models resulting from three repeats of five-fold stratified cross-validation are shown (Fig. 2). Among these algorithms, the XGBoost model achieved the highest mean (SD) AUROC of 0.87 [0.06] with a recall of 0.78 and a precision of 0.79. Therefore, we selected XGBoost algorithm to build prediction models.

### Feature Importance analysis and development of 4-Elements model

**Figure 3:**
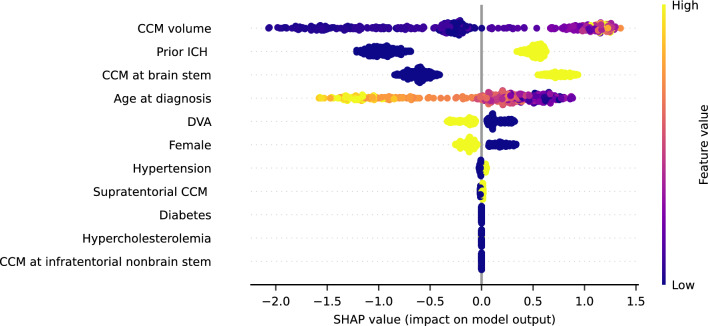
Summary plot of SHAP (SHapley Additive exPlanations) based on XGBoost model. The plot shows the ranking of all 11 features affecting the output of the XGBoost model. Each point in every feature is on behalf of a concrete sporadic CCM patient, with a Shapley value for the respective feature. Feature importance is ranked by the sum of Shapley values in a descending manner. *CCM* cerebral cavernous malformations, *ICH* intracerebral hemorrhage, *DVA* developmental venous anomaly.

To shed light on the feature importance, Shapley values based on the XGBoost model were calculated. The feature importance ranking, determined by the sum of the Shapley value magnitudes, is illustrated in Fig. 3, each point with color on behalf of the feature value of one patient.

For easy usage of prediction models for clinicians, the 4-Elements model based on the top 4 features (CCM volume, prior ICH, CCM at brain stem, age at diagnosis) using XGBoost was built. It should be noted that, for those sporadic CCM patients with prior ICH, CCM volume was measured as the sum of CCM lesion and hemorrhage lesion. The ROC curves of 4-Elements model in cross-validation are demonstrated in Supplementary Fig. S1.

### Performance of all-Elements model and 4-Elements model on testing cohort

**Figure 4:**
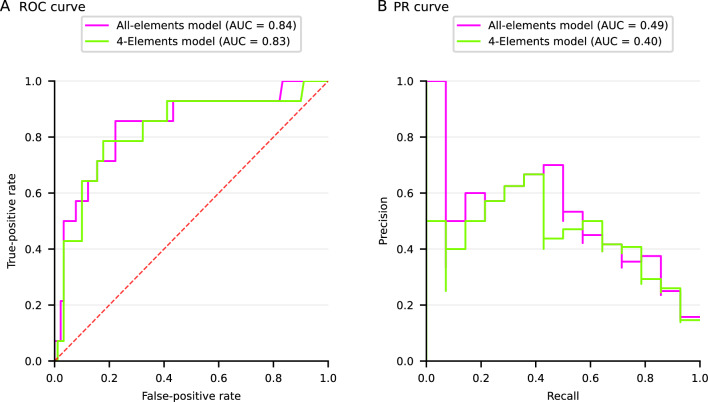
The ROC curves and precision recall curves for all-Elements model and 4-Elements model on the testing set. ROC curves (**A**) and precision recall curves (**B**) for evaluating all-Elements model and 4-Elements model on the testing set. ROC curves are receiver operating characteristic curves. *AUC* area under the curves.

Figure 4 shows ROC curves and PR curves of all-Elements model and 4-Elements model for testing cohort. The all-Elements model generated AUROCs of 0.84, whereas this value for 4-Elements model was 0.83. The all-Elements model and 4-Elements model demonstrated a PR-AUC of 0.49 and 0.40, respectively.

Table 2 demonstrates the representative performance of developed models. The sensitivity and specificity of all-Elements model based on XGBoost were 0.86 and 0.76. 4-Elements model achieved a sensitivity of 0.79 and a specificity of 0.72.

**Table 2:** The performance of all-Elements model and 4-Elements model on testing cohort.

	Sensitivity	Specificity	PPV	NPV	LR +	LR−
XGBoost						
All-Elements model	0.86	0.76	0.35	0.97	3.51	0.19
4-Elements model	0.79	0.72	0.31	0.96	2.83	0.30

*PPV* positive predictive value, *NPV* negative predictive value, *LR* + positive likelihood ratio, *LR–* negative likelihood ratio.

## Discussion

To the best of our knowledge, the all-Elements model and 4-Elements model are the first developed machine-learning based models for detecting the potential (re)hemorrhage among sporadic CCM patients, especially the readily used 4-Elements model. The present developed all-Elements model using XGBoost algorithm achieved an AUROC of 0.84, with a sensitivity of 0.86 and a specificity of 0.76, demonstrating a comparatively accurate performance in identifying the potential (re)hemorrhage among CCM patients within 5 years. Importantly, the 4-Elements model yielded accurate performance as well in detecting the potential (re)hemorrhage, with an AUROC of 0.83, a sensitivity of 0.79, and a specificity of 0.72.

Compared to SVM model and stacking model, the developed XGBoost model yields higher AUROC in predicting the potential (re)hemorrhage using 11 clinical records of CCM patients. Shapley values have been adopted to interpret feature importance^20,22,29,38^ and feature importance based on the XGBoost model was ranked with the aid of Shapley values. For easy and ready usage in clinical practice, previous studies have built machine learning models using only several top features^30,39,40^. For our XGBoost model, the top 4 features are CCM volume, presence of ICH, CCM at brain stem, and age at diagnosis, with which we try to build 4-Elements model. Researchers identified prior hemorrhage as a major risk factor for subsequent hemorrhage^7,9,41–43^. Localized in deep regions of the brain, brainstem CCM and thalamic CCM took up approximately one-third^44^ and it was found that CCM lesions at the brainstem increased hemorrhage rate^12,45–48^.

Abundant evidence links age with the risk of (re)hemorrhage among CCM patients. Based on 242 patients with brainstem CCM, Li et al. found that the interval of rehemorrhage-free was significantly shorter in patients aged 50 years or older^49^ and subsequent studies also showed that patients aged 55 years or older were associated with hemorrhage^50^. However, the finding related to the role of age in (re)hemorrhage of CCM patients is not consistent. Young age (< 40 years or < 45 years) was suggested to be associated with CM hemorrhage^13,51^. In contrast with the above conclusion, several studies also demonstrated that age was not associated with subsequent symptomatic hemorrhage among CCM patients^47,52^. From the sight based on machine learning, we identified age at diagnosis of CCM as a risk factor for (re)hemorrhage.

Although decades of surgical excision for CCM patients, surgical treatment remains controversial^4^. Neurosurgical excision of CCM is executed to prevent symptomatic ICH and the risk of CCM resection includes death or nonfatal stroke^4,53^. To prevent potential hemorrhage, surgical excision could be considered in asymptomatic CCM patients in noneloquent areas^54^. CMs located in proximity to the ventricular system or easily accessible solitary CMs in non-eloquent areas may be in need of neurosurgical treatment^55^. Surgery for CCMs at critical supratentorial areas caused significant mainly transient morbidity, and these could be recovered over time^56^. Performing surgery in a subacute phase 2–4 weeks after bleeding is suggested for CCM patients^55^.

It is worth noting that several findings concluded CCM size was not a risk factor for the hemorrhage rate^47,51,52,57,58^. However, one study based on anatomical location also found that CCM with volume (≥ 1 cm^3^) at infratentorial cavernous lesions was associated with a high risk of CM rupture whereas that at supratentorial cavernous lesions did not show any relating sign^13^. It is clearly shown that all the top 4 features interpreted by Shapley values have been suggested to be associated with CCM hemorrhage, which may ensure the accurate performance of our 4-Elements model. Interestingly, CCM volume in our prediction model has been demonstrated as the top 1 feature for distinguishing potential (re)hemorrhage of CCM patients from those without (re)hemorrhage risk. We suppose the underlying mechanism may be that machine learning algorithms do not view solitary features and complex relationships between features significantly influencing the resulting classification may be constructed^22^ in the process of building a model. CCM volume in this study was measured by the sum of CCM lesion and hemorrhage lesion in the case of CCM patients with prior ICH^15^.

Although we impute missing values in features of hypercholesterolemia, hypertension and diabetes, these features weakly affect the output of XGBoost model by viewing Shapley values. Further, features used to build the 4-Elements model do not contain missing values.

Surgical resection is a definitive cure for selected CCM patients though remains conflicting due to substantial operative risks^3,4^. Antithrombotic therapy use in a long-term lowered the risk of ICH in CCM patients^19^. CCM patients labeled by the two machine learning models as potential (re)hemorrhage should, therefore, be considered by clinicians as requiring prompt treatment. Further, CCM patients who are predicted by two models without risk of potential (re)hemorrhage could avoid unnecessary treatment. Our all-Elements model and 4-Elements model fill the gap that the potential (re)hemorrhage CCM patients within 5 years among CCM patients could be recognized in advance.

This study has inherent limitations. 517 sporadic CCM patients were included in this study, and large datasets may need to further validate the all-Elements model and 4-Elements model. Moreover, collecting sufficient clinical records of sporadic CCM patients may facilitate select important features greatly influencing the output of the model.

In conclusion, we developed all-Elements XGBoost model and 4-Elements XGBoost model for identifying potential (re)hemorrhage within 5 years among sporadic CCM patients, both achieving comparatively accurate performance. Importantly, the 4-Elements model is convenient for clinical usage. The two models will aid clinical decision-making, such as initiating prompt treatment for the potential (re)hemorrhage or avoiding unnecessary treatment for those without (re)hemorrhage risk. We are limited by the size of institutions to collect follow-up data of CCM patients for external validation, and we also could not find a dataset of CCM patients in the open platform. Further validating the all-Elements model and 4-Elements model with large datasets of sporadic CCM patients is necessary.

### Supplementary Information

Supplementary Information.

## Data Availability

The data and code supporting this study is available from the corresponding author upon reasonable request.
