# Supplementary material for: Identifying potential (re)hemorrhage among sporadic cerebral cavernous malformations using machine learning
Source: Sci Rep. 2024 May 14;14:11022. doi: 10.1038/s41598-024-61851-4 (PMC11094099; doi:10.1038/s41598-024-61851-4)

**Supplementary Material.**

Table S1: Search Space of Hyperparameters and Optimal Results for SVM Model, Stacking Model, All-Elements XGBoost Model, and 4-Elements XGBoost Model`

| **Algorithm** | **Hyperparameter** | **Search Space** | **Optimal** |
| --- | --- | --- | --- |
| SVM |  |  |  |
|  | kernel | “rbf”, “sigmoid” | “rbf” |
|  | gamma | “scale”, “auto”, 0.01, 0.1, 1, 10,100 | “scale” |
|  | C | 0.01, 0.1, 1, 10, 100 | 100 |
| Stacking |  |  |  |
| SVM | kernel | “rbf”, “sigmoid” | “rbf” |
|  | C | 0.01, 0.1, 1, 10, 100 | 10 |
| RFC | n_estimators | 50, 60, 100, 200 | 100 |
|  | criterion | “gini”, “entropy” | “gini” |
| GBDT | n_estimators | 50, 100, 300 | 300 |
|  | learning_rate | 0.01, 0.1, 0.25, 1 | 0.25 |
| Logistic regression | solver | “newton-cg”, “lbfgs”, “liblinear” | “newton-cg” |
| XGBoost |  |  |  |
| All-Elements model |  |  |  |
|  | n_estimators | 400, 500, 600, 800,1000 | 600 |
|  | learning_rate | 0.0001, 0.001, 0.01, 0.1, 1 | 0.01 |
|  | max_depth | 6, 7, 8, 10 | 6 |
|  | gamma | 0, 0.2, 0.4, 0.6, 0.8, 1 | 1 |
|  | subsample | 0.6, 0.7, 0.8, 0.9, 1 | 0.6 |
|  | colsample_bytree | 0.6, 0.7, 0.8, 0.9, 1 | 1 |
| 4-Elements model |  |  |  |
|  | n_estimators | 100, 400, 500, 600 | 100 |
|  | learning_rate | 0.0001, 0.001, 0.01, 0.1, 0.3, 0.4, 1 | 0.0001 |
|  | max_depth | 6, 7, 8, 10 | 6 |
|  | gamma | 0, 0.2, 0.4, 0.6, 0.8, 1 | 0.2 |
|  | subsample | 0, 0.2, 0.4, 0.6, 0.8, 1 | 0.5 |
|  | colsample_bytree | 0.1, 0.3, 0.4, 0.6, 0.7, 0.9, 1 | 0.6 |

Figure S1: ROC Curves of 4-Elements Model in the Cross-Validation


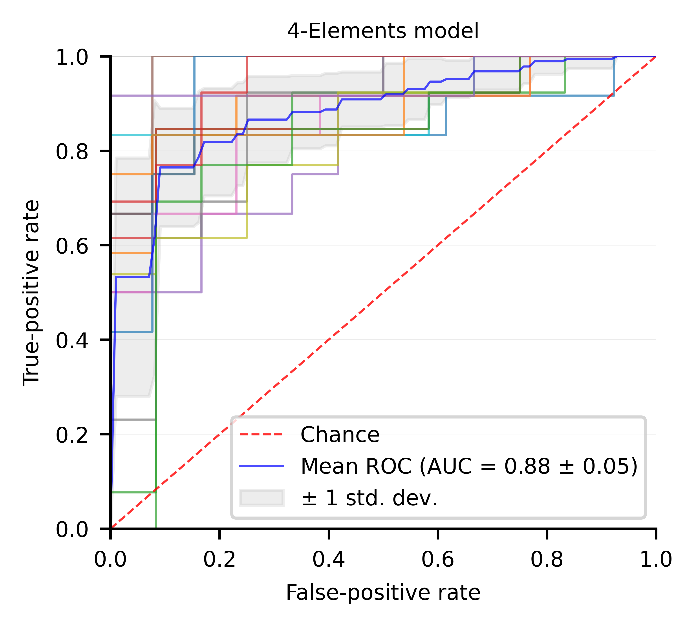

Supplement: Supplementary file 1 — Supplementary Information. [file 41598_2024_61851_MOESM1_ESM.docx]
